# Differential expression in humans of the viral entry receptor ACE2 compared with the short *delta*ACE2 isoform lacking SARS-CoV-2 binding sites

**DOI:** 10.1038/s41598-021-03731-9

**Published:** 2021-12-21

**Authors:** Thomas L. Williams, Gregory Strachan, Robyn G. C. Macrae, Rhoda E. Kuc, Duuamene Nyimanu, Anna L. Paterson, Sanjay Sinha, Janet J. Maguire, Anthony P. Davenport

**Affiliations:** 1 Experimental Medicine and Immunotherapeutics, University of Cambridge, Level 6, Addenbrooke’s Centre for Clinical Investigation, Addenbrooke’s Hospital, Box 110, Cambridge, CB2 0QQ UK; 2 Wellcome Trust-MRC Institute of Metabolic Science, Metabolic Research Laboratories, Addenbrooke’s Biomedical Campus, Cambridge, UK; 3 Wellcome-MRC Cambridge Stem Cell Institute, Jeffrey Cheah Biomedical Centre, University of Cambridge, Cambridge, UK; 4 Department of Pathology, Royal Papworth Hospital NHS Foundation Trust, Cambridge University Hospitals NHS Foundation Trust, Cambridge, UK

**Keywords:** Medical research, Translational research

## Abstract

ACE2 is a membrane protein that regulates the cardiovascular system. Additionally, ACE2 acts as a receptor for host cell infection by human coronaviruses, including SARS-CoV-2 that emerged as the cause of the on-going COVID-19 pandemic and has brought unprecedented burden to economy and health. ACE2 binds the spike protein of SARS-CoV-2 with high affinity and shows little variation in amino acid sequence meaning natural resistance is rare. The discovery of a novel short ACE2 isoform (*delta*ACE2) provides evidence for inter-individual differences in SARS-CoV-2 susceptibility and severity, and likelihood of developing subsequent ‘Long COVID’. Critically, *delta*ACE2 loses SARS-CoV-2 spike protein binding sites in the extracellular domain, and is predicted to confer reduced susceptibility to viral infection. We aimed to assess the differential expression of full-length ACE2 versus *delta*ACE2 in a panel of human tissues (kidney, heart, lung, and liver) that are implicated in COVID-19, and confirm ACE2 protein in these tissues. Using dual antibody staining, we show that *delta*ACE2 localises, and is enriched, in lung airway epithelia and bile duct epithelia in the liver. Finally, we also confirm that a fluorescently tagged SARS-CoV-2 spike protein monomer shows low binding at lung and bile duct epithelia where dACE2 is enriched.

## Introduction

The *ACE2* gene, mapped to chromosome locus Xp22.2, encodes angiotensin-converting enzyme 2 (ACE2, UniProt ID: Q9BYF1), comprising 805 amino acid residues (~ 120 kDa mass) and functioning as a zinc-metalloproteinase type 1 transmembrane protein^1–4^. ACE2 is a critical regulator of the renin–angiotensin-system in the cardiovascular system, where it counteracts increases in blood pressure through metabolism of angiotensin peptides^3,5–7^. Additionally, ACE2 has been shown to cleave the apelin peptide [Pyr^1^]apelin-13 to [Pyr^1^]apelin-13_(1–12)_, which is expressed and functional as a potent vasoactive agent and positive cardiac inotrope in the cardiovascular system^8^. ACE2 also acts as a chaperone for epithelial and brush-border expression of the neutral amino acid transporter, B^0^AT1 (*SLC6A19*), in proximal tubules of the kidney and the small intestine respectively^9–11^.

Surprisingly, ACE2 is exploited as a host cell surface receptor for entry of viruses such as the severe acute respiratory syndrome coronavirus (SARS-CoV) and human corona virus NL63 (HCoV-NL63), where ACE2 has been shown to efficiently bind the S1 domain of coronavirus spike (S) proteins^3,12–14^. The ongoing corona virus disease 2019 (COVID-19) pandemic, that has put huge strain on health care services across the globe, is caused by severe acute respiratory syndrome coronavirus 2 (SARS-CoV-2). As is the case for SARS-CoV and HCoV-NL63, ACE2 is able to bind the SARS-CoV-2 spike (S) protein with high affinity (~ 15 nM) to facilitate host cell infection, and remains a critical therapeutic target that may need to be exploited to combat the virus and its emerging variants^15–19^. ACE2, long seen as a beneficial protein in the protective arm of the renin-angiotensin-system, is therefore a negative mediator of viral infection that has been reviewed extensively in the COVID-19 pandemic^20–22^. Interestingly, the ACE2-B^0^AT1 heterodimer complex was shown using cryogenic electron microscopy to be able to bind two SARS-CoV-2 spike proteins simultaneously^16^. Recently, both ACE2 and B^0^AT1 have been targeted pharmacologically, with the compounds DX600 and benztropine respectively, to reduce SARS-CoV-2 spike dependent viral infection in a human embryonic stem cell derived cardiomyocyte model^23^.

Structural modelling identifies several key residues in the extracellular domain of ACE2 that interact with SARS-CoV-2 spike protein^17^. The authors suggest that Q474, Q498, T500, and N501 of the receptor binding domain of spike forms a network of H-bonds with Q24, Y41, Q42, K353, and R357 of ACE2. K417 and Y453 of the spike receptor binding domain also interact with D30 and H34 of ACE2. F486 of the spike protein may also interact with M82 through van der Waals forces. Due to a lack of selection pressure prior to the COVID-19 pandemic, ACE2 population variants are surprisingly rare^4,24^. Several ACE2 polymorphisms have been identified that are associated with hypertension^25–28^, but it remains unclear whether these have an impact on SARS-CoV-2 interaction. Several genomic studies confirm few natural resistance mutations in ACE2 exist, with many of the identified variants exhibiting similar binding affinity for SARS-CoV-2 spike and some ACE2 variants (such as I21V, E23K, K26R, T27A, N64K, T92I, Q102P, and H378R) that are even predicted to confer increased susceptibility to SARS-CoV-2^24,29^. Interestingly however, the alternate allele (T or A) of ACE2 rs2285666 correlated with lower infection and case-fatality rate among Indian populations^30^ and ACE2 alleles rs73635825 (S19P) and rs143936283 (E329G) showed noticeable variations in their intermolecular interactions with the viral spike protein^29^. Host cell receptor variation, such as the Δ32 variant in C–C chemokine receptor type 5 (CCR5) that confers resistance to strains of HIV^31^, is a critical concept in understanding viral entry and pharmacological intervention—and ACE2 variation will need to be studied further in relation to SARS-CoV-2 infection.

ACE2 shows relatively wide tissue distribution. In original work, particularly high mRNA expression was observed in the kidney, testis, and heart^1,2^, where ACE2 was localised to the vascular endothelium, smooth muscle, myofibroblasts, and the myocytes themselves^32,33^. QRT-PCR performed in 72 tissue types confirmed expression in cardiovascular tissues but also showed high abundance of mRNA in the gastrointestinal system, particularly the intestines^34^. Protein expression has been identified in the vascular endothelium and smooth muscle, lung alveolar epithelial cells, intestinal enterocytes^35^, and also in cardiomyocytes, gall bladder, and renal tubules^36^. Interestingly, expression in the respiratory tract has been shown to be relatively low overall, and restricted to certain structures and subsets of cells—chiefly cells of the sinonasal cavity and alveolar type II cells^35–38^. The localisation of ACE2 at nasopharyngeal, lung, and gastrointestinal tract epithelia falls in line with the proposed entry routes for SARS-CoV-2 infection^37,39–41^. Importantly, as ACE2 resides in the X chromosome, females show higher overall expression or ‘gene dosing’ of the protein^42^, which paradoxically may contribute to the reduced susceptibility to SARS-CoV-2 symptoms and mortality versus males observed globally in COVID-19 cases^43,44^.

To date, two recent reports provide further insight into the spectrum of responses of individuals, ranging from those that are asymptomatic, to those with severe illness and long-term effects such as Long COVID that affects the heart. The papers describe a novel isoform *delta*ACE2 (herein referred to as dACE2) that is upregulated by interferon stimulation and rhinovirus infection but not SARS-CoV-2^45,46^. This short isoform, comprising amino acids 357–805 of ACE2 with a 10 amino acid insert at the N-terminus, lacks both a fully functional enzyme catalytic site and high affinity spike S1 binding sites, and is observed in airway epithelia and squamous tumours of the respiratory, gastrointestinal, and urogenital tracts. Both studies conclude the short isoform is unlikely to confer host susceptibility to infection by SARS-CoV-2, and that it is this isoform, not regular ACE2, that is upregulated in response to interferon or rhinovirus infection. Whilst these studies focused primarily on ACE2 and dACE2 mRNA, the protein distribution has not been extensively mapped.

We hypothesise that the tissue distribution of the full length ACE2 versus non-virus binding dACE2 will be distinct between organs and specific tissue beds, and may give insight as to why susceptibility to infection varies. Our aim was to use immunohistochemistry to compare the protein distribution of the ACE2 isoforms in the major peripheral organs in humans that show SARS-CoV-2 dependent viral damage in post-mortem assessment. We find both isoforms are localised in multiple tissue types in humans but protein expression of dACE2 is enriched in the epithelial cells of the lungs and bile duct. We speculate that these tissues could be used as systems to identify mechanisms that alter short-term dACE2 expression and, more widely, potentially reduce infection. Additionally, we confirm low binding of a fluorescently tagged SARS-CoV-2 spike protein monomer in lung airway and bile duct epithelia where dACE2 was enriched.

## Results

### Full-length ACE2 protein is expressed in human organs associated with SARS-CoV-2 infection and symptoms

**Figure 1:**
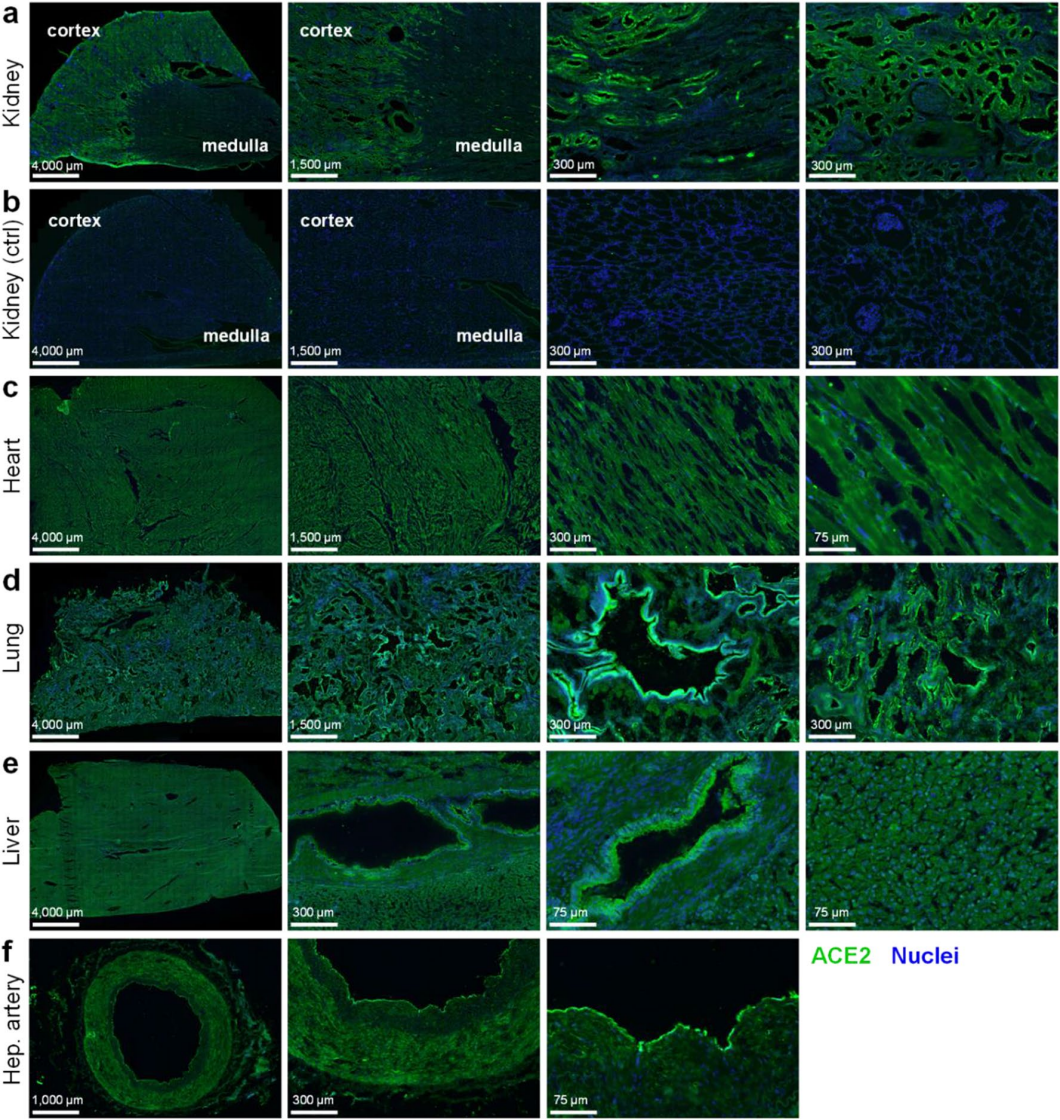
Immunohistochemistry confirms positive staining for ACE2 in several human tissue types. Representative fluorescent images of 4% formaldehyde fixed human tissue sections (n ≥ 3 independent donors, stained in duplicate) treated with ACE2*poly* antibody (R&D AF933), visualised in green, and Hoechst 33342 nuclear stain, visualised in blue. Scale bars are as indicated in figure. Tissues shown include: (**a**) Kidney, with cortex and medulla indicated; (**b**) Kidney control section treated with secondary antibody and Hoechst 33342 alone; (**c**) Heart tissue, comprised predominantly of cardiomyocytes; (**d**) Lung, with preserved airway structures; (**e**) Liver, comprised predominantly of hepatocytes with preserved bile duct structures; (**f**) Hepatic artery section.

We confirm by immunohistochemistry the presence of ACE2 protein in a panel of human tissues known to be infected and/or damaged by SARS-CoV-2 (Fig. 1a–f). The specificity of the antibodies used, and criteria for quantitatively measuring fluorescence to assess specific staining over background is described in detail in Supplementary Information.

In tissue sections, a polyclonal ACE2 antibody (R&D AF933; herein referred to as ACE2*poly*) directed against multiple epitopes of the extracellular domain of ACE2 protein, was used with a secondary antibody to visualise ACE2 distribution (shown in green). In kidney (Fig. 1a), strong positive staining was observed in the tubules of the renal cortex, with less staining observed in the glomeruli, and tubules of the renal medulla. Control kidney sections (Fig. 1b), treated with secondary antibody in the absence of ACE2*poly* and imaged using identical illumination settings shows very low staining, indicating the requirement of the ACE2*poly* primary antibody for positive, specific signal. In the heart, specifically the left ventricle (Fig. 1c), strong ACE2 staining was present across the cardiomyocyte population that makes up the bulk of the tissue. In the lung (Fig. 1d), well established as one of the primary sites of SARS-CoV-2 infection, strong ACE2 staining across the tissue was particularly bright in airway epithelia. The liver (Fig. 1e) showed low levels of ACE2 staining across the hepatocyte population, with noticeably stronger staining observed in the bile duct epithelium. Finally, staining was also observed in hepatic artery sections (Fig. 1f), with highest fluorescence signal seen in the endothelium.

### Short dACE2 protein is not enriched in renal or cardiac tissue

Following the recent discovery of the short dACE2 isoform, we performed further immunohistochemical experiments using the aforementioned ACE2*poly* antibody, in conjunction with a monoclonal ACE2 antibody (ab108252; herein referred to as ACE2*mono*). ACE2*mono* is raised against a single immunogen of ACE2 between residues 200–300 in the extracellular domain of ACE2 that is absent in dACE2. The two antibodies have been used in other studies to distinguish between the full-length and short ACE2 proteins, and we aimed to see whether discrepancies in the binding of the two antibodies are observed in tissue substructures that have not been examined previously.

**Figure 2:**
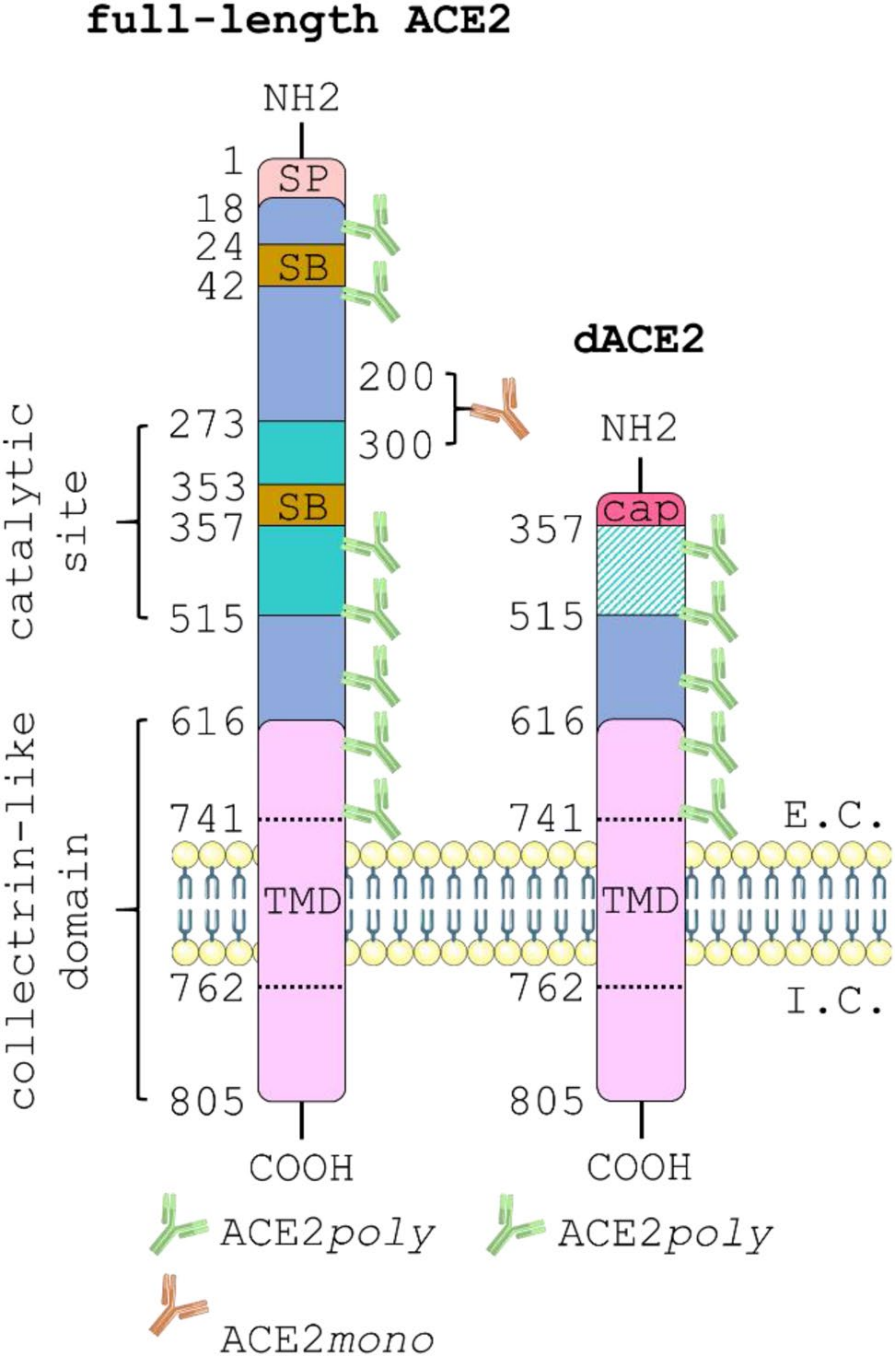
Schematic showing the critical protein domains of full-length ACE2 versus the short dACE2 isoform. The 805 amino acid full-length ACE2 protein (left) is comprised of an extracellular domain that protrudes into the extracellular (E.C.) space and an intracellular domain that remains in the intracellular (I.C.) space. The extracellular domain is made up of a signal peptide (SP) that extends from positions 1–18; the peptide-binding catalytic site that covers 272–515; two spike protein binding sites (SB) located at 24–42 and 353–357; a collectrin-like domain (CLD) that covers 616–805; and a short transmembrane domain (TMD) that spans the membrane at positions 741–762. The short dACE2 isoform (right) loses all amino acids up to positon 357 and a unique 10 amino acid sequence caps the N-terminus. Note that dACE2 has lost both its spike protein binding sites and the catalytic site is non-functional. The diagram also shows the potential binding sites for the ACE2*poly* antibody (green), raised against an 18–740 amino acid immunogen of ACE2, versus the single proprietary binding site that sits between amino acids 200–300 for the ACE2*mono* antibody (orange). If the full ACE2 isoform is present, green and orange fluorescent signal will be observed in immunological staining studies. If only the short ACE2 isoform is present, green fluorescent signal alone will be observed due to the lack of the monoclonal antibody binding site. The schematic was generated using templates from Servier Medical Art (smart.servier.com).

Figure 2 shows a schematic outlining the key protein domains and antibody binding sites of full-length ACE2 versus dACE2. As indicated, colocalisation of ACE2*poly* (visualised in green) with ACE2*mono* (visualised in orange) is hypothesised to show the presence of full-length ACE2, whilst observation of ACE2*poly* in the absence of ACE2*mono* suggests that only the short isoform is present.

**Figure 3:**
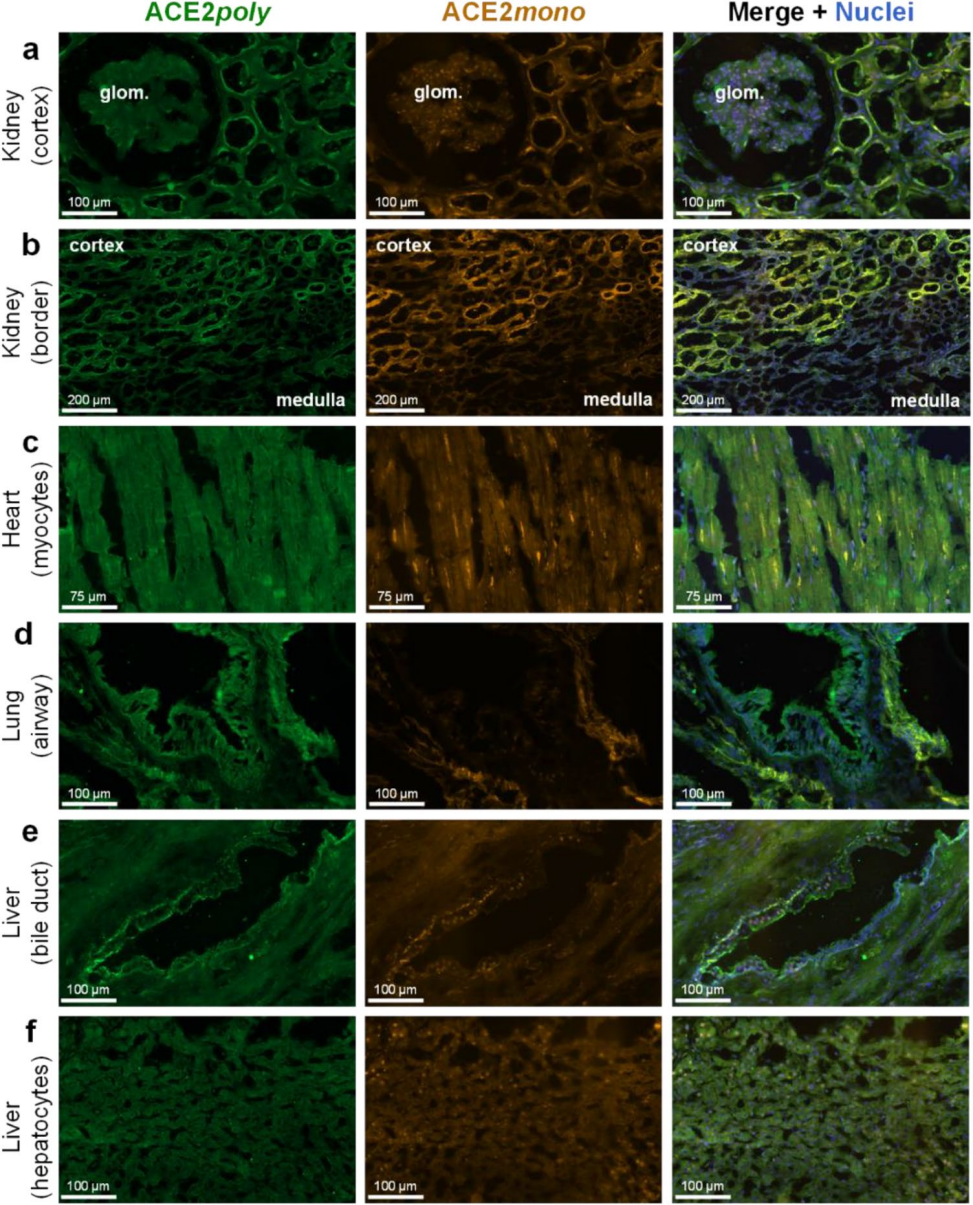
Differential expression of full-length ACE2 and the short dACE2 isoform in a panel of human tissues. Representative fluorescent images (n = 3 independent donors, stained in duplicate) of human tissue fixed in 4% formaldehyde and treated with ACE2*poly* (left column, visualised in green) and ACE2*mono* (centre column, visualised in orange). Merged images (right column, overlay) also include Hoechst 33342 nuclear marker (visualised in blue). Scale bars are as indicated in figure. Tissues shown include: (**a**) Kidney cortex, with a glomerulus (glom.) indicated; (**b**) Kidney border, showing a region where tubules of the cortex meet tubules of the medulla; (**c**) Heart tissue, showing cardiomyocytes; (**d**) Lung tissue, showing an airway structure; (**e**) Liver bile duct; (**f**) Liver hepatocytes.

In the kidney, both ACE2*poly* and ACE2*mono* showed strong positive staining that colocalised in epithelial cells lining the tubules of the renal cortex (Fig. 3a), with lower levels observed in the glomerulus in accordance with previous findings in the literature. At the kidney border (Fig. 3b) where the renal cortex meets the renal medulla, the staining with both antibodies is lower in the tubules of the medulla. Overall, immunohistochemistry in the kidney showed good colocalisation of ACE2*poly* with ACE2*mono* suggesting that the ACE2 present in this tissue type is predominantly the full-length protein. Cardiomyocytes in the left ventricle of the heart (Fig. 3c) also show positive ACE2 staining with both antibodies, again suggesting that the full-length isoform is present in this tissue.

### Short dACE2 protein enrichment in the lung and liver is restricted to epithelial cells

In the lung (Fig. 3d), epithelial cells of airway structures showed strong positive staining with ACE2*poly* whilst staining with ACE2*mono* in these structures was observed to a substantially lesser extent, and in some cases was almost completely absent. This is in agreement with previous findings that show expression of dACE2 mRNA in lung and differentiated airway cell line. Staining with the two antibodies in other areas of the lung, such as blood vessels and connective tissue, showed good colocalisation, suggesting that it is predominantly the respiratory epithelial cells of airways that show differential expression of the two ACE2 isoforms.

Liver expression also showed differential patterns of staining with ACE2*poly* and ACE2*mono*. We report, for the first time, that epithelial cells lining bile duct structures exhibited particularly strong staining with ACE2*poly* that did not colocalise with ACE2*mono*, providing evidence for the presence of dACE2 in this tissue bed (Fig. 3e). In hepatocytes (Fig. 3f), signal with both antibodies was low but colocalised well, as seen in the merge, indicating that only the epithelial cells of the bile duct exhibit differential expression of ACE2 isoforms.

**Figure 4:**
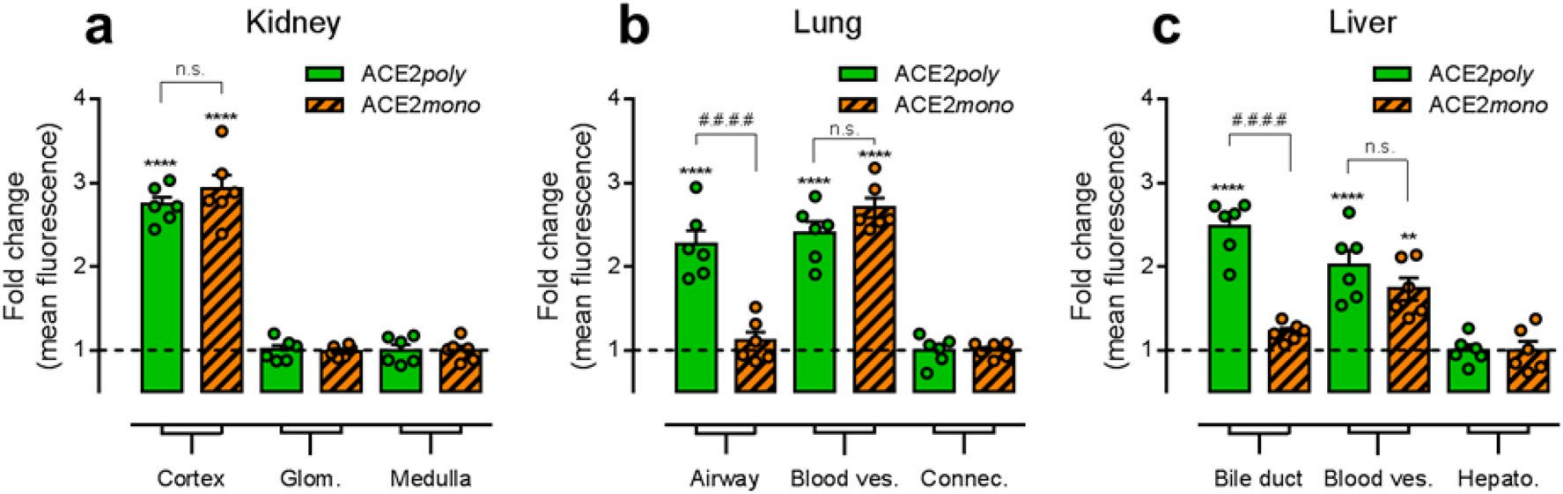
Quantification of differential expression of full-length ACE2 and the short dACE2 isoform in specific structures of human tissues. Graphical output shows the fold change in mean fluorescence observed for the respective secondary antibodies used to visualise ACE2*poly* and ACE2*mono* in distinct anatomical structures of the human tissue sections shown in Fig. 3. For each structure, n = 6 from ≥ 2 tissue sections. (**a**) Fold change in mean fluorescence observed for ACE2*poly* and ACE2*mono* in kidney cortex and glomeruli (Glom.), versus the normalised mean fluorescence observed in the renal medulla. (**b**) Fold change in mean fluorescence observed for ACE2*poly* and ACE2*mono* in airway structures and blood vessels (Blood ves.) of the lung, versus the normalised mean fluorescence observed in the connective tissue (Connec.). (**c**) Fold change in mean fluorescence observed for ACE2*poly* and ACE2*mono* in liver bile ducts and blood vessels (Blood ves.), versus the normalised mean fluorescence observed in liver hepatocytes (Hepato.). All data show mean ± SEM with individual data points shown. Statistical analyses of data included a one way ANOVA with multiple comparisons using Tukey’s correction. Statistical significance was determined where p < 0.05. **** or ^####^ = p < 0.0001; ** = p < 0.01; n.s. = no significant difference.

Quantification (see Supplementary Figs. 1 and 2 for methodological approach) of the fold change in mean fluorescence intensity in distinct tissue substructures and cell types (Fig. 4a–c) closely matched the qualitative observations in the tested human tissue. In the kidney, a significant increase in fold change in mean intensity of ACE2*poly* fluorescence of 2.74 ± 0.09 was observed in the tubules of the cortex versus the medulla which was normalised to 1.00 ± 0.07 (Fig. 4a). Unlike the cortical tubules, the fold change for glomerular structures (1.02 ± 0.05) was not significantly different from the distal tubules (Fig. 4a), closely matching the qualitative assessment in Fig. 3a,b). The ACE2*mono* fold change was also only significantly higher in the cortical tubules (2.93 ± 0.17) and not glomerular structures (0.99 ± 0.03) versus the respective, normalised fold change for this antibody observed in the medulla (1.00 ± 0.05) (Fig. 4a). No significant difference was determined for the fold change in ACE2*poly* and ACE2*mono* fluorescence in the cortical tubules themselves. Overall, data in the kidney confirms a high abundance of full-length ACE2 protein in the tubules of the renal cortex, with lower expression in the glomeruli, and tubules of the renal medulla.

In the lung (Fig. 4b), fold change in mean intensity for ACE2*poly* fluorescence was significantly higher in airway structures (2.26 ± 0.17) than the normalised fold change observed for this antibody in connective tissue (1.00 ± 0.07). Conversely, the fold change for the ACE2*mono* (1.11 ± 0.11) was not significantly different to the normalised fold change observed for this respective antibody in connective tissue (1.00 ± 0.04), indicating that, while ACE2 is enriched in airways, as detected by increased ACE2*poly*, we observe enrichment of dACE2, and not the full-length isoform that is stained by ACE2*mono*. In confirmation, the fold change for ACE2*poly* in airways was significantly higher than the fold change for ACE2*mono*. Blood vessels in the lung showed significant, high enrichment with both ACE2*poly* (fold change of 2.40 ± 0.14) and ACE2*mono* (fold change of 2.70 ± 0.12) against the respective, normalised connective tissue fold changes, indicating that these substructures do not show enrichment purely of dACE2, but with both isoforms. There was no significant difference in fold change in fluorescence between the two antibodies in the lung blood vessels. In summary, in lung tissue we observed enrichment of dACE2 in airways, with no change in the full-length isoform when compared to the staining observed in the connective tissue. This agrees with previous findings and our own qualitative assessment in Fig. 3d.

In the liver (Fig. 4c), the bile duct showed a significantly higher fold change in mean fluorescence with the ACE2*poly* (2.48 ± 0.13) versus the normalised fold change observed with this antibody in hepatocytes (1.00 ± 0.07). This was not the case again for the ACE2*mono*, where a fold change of 1.22 ± 0.04 was not significantly different to the normalised fold change for this antibody in hepatocytes (1.00 ± 0.10). Like the airways in the lung, this suggests that there is enrichment of ACE2 in the bile duct above the levels of ACE2 observed in hepatocytes, but it is predominantly dACE2 that sees enrichment over the regular isoform. In direct comparison, fold change in mean fluorescence with ACE2*poly* was significantly higher than that for ACE2*mono*. As was also seen in the lung, blood vessels in the liver saw significant fold changes in mean fluorescence with the ACE2*poly* (2.02 ± 0.17) and ACE2*mono* (1.73 ± 0.13) against the respective, normalised hepatocyte fold changes, indicating again that these substructures likely show enrichment with both isoforms of ACE2. Our qualitative and quantitative assessment of liver tissue suggests an epithelial enrichment of the short dACE2 isoform in the bile duct that is similar to that seen for epithelial cells in the airway of the lung.

### SARS-CoV-2 spike protein shows low binding at tissue structures associated with enriched dACE2

**Figure 5:**
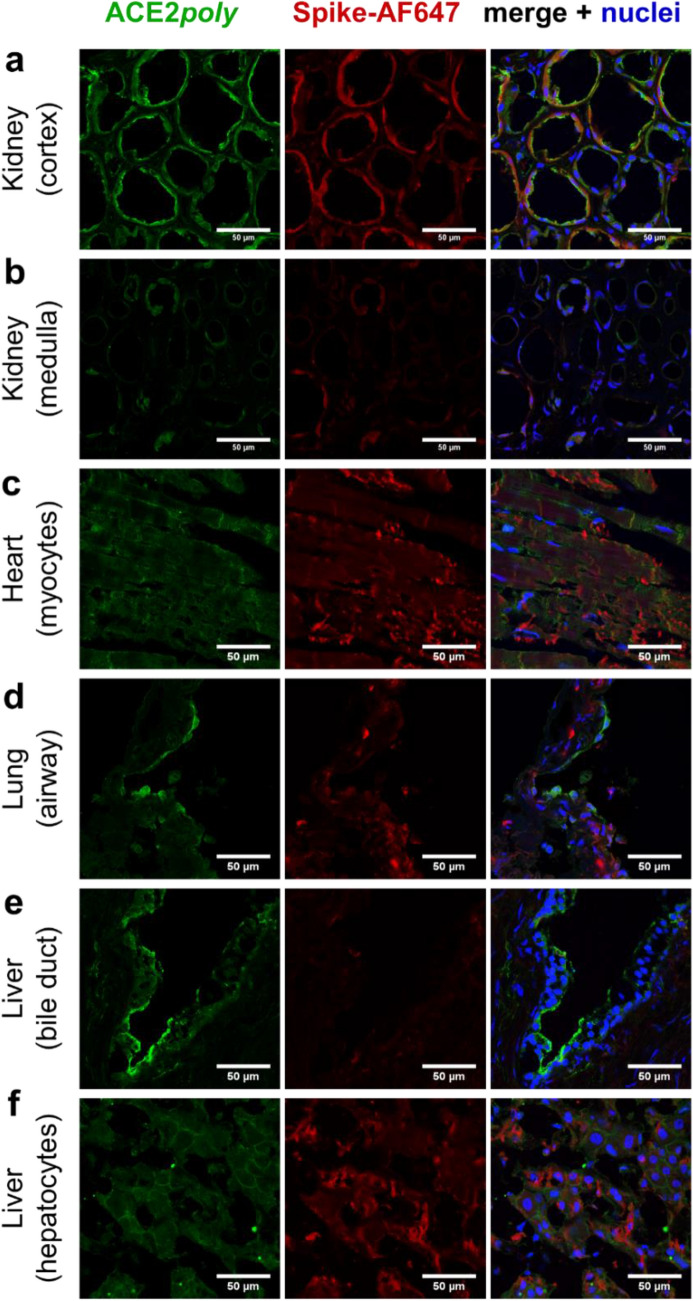
Binding of fluorescent SARS-CoV-2 spike-AF647 in ACE2 positive cells in a panel of human tissues. Representative confocal fluorescent images (n ≥ 2 independent donors, stained in duplicate) of human tissue fixed in 4% formaldehyde and treated with ACE2*poly* (left column, visualised in green) and 1 µM spike-AF647 (centre column, visualised in red). Merged images (right column, overlay) also include Hoechst 33342 nuclear marker (visualised in blue). Scale bars are as indicated in figure. Tissues shown include: (**a**) Kidney cortex; (**b**) Kidney medulla; (**c**) Left ventricle heart tissue, showing cardiomyocytes; (**d**) Lung tissue, showing an airway structure; (**e**) Liver bile duct; (**f**) Liver hepatocytes.

Finally, we used a fluorescently tagged SARS-CoV-2 spike receptor binding motif protein monomer (herein referred to as spike-AF647; 1 µM) to assess binding to ACE2 (Fig. 5) in the panel of aforementioned human tissues. Fluorescent confocal images of human kidney sections (Fig. 5a) displayed signal for ACE2*poly* (visualised in green) and spike-AF647 (visualised in red) that localised to tubules in the renal cortex, whilst signal for both fluorophores was low to absent in the renal medulla (Fig. 5b). Cardiomyocytes in left ventricle tissue sections (Fig. 5c) also showed good signal with both ACE2*poly* and spike-AF647 across the cell population.

Intriguingly, lung tissue (Fig. 5d) showed very strong ACE*poly* signal in airway structures, but spike-AF647 showed comparatively low signal, particularly at the epithelial edge. This matched our findings in Figs. 3d and 4b where dACE2, that lacks SARS-CoV-2 spike binding sites, was enriched at the epithelial edge of airway structures. Additionally, we also observed ACE2*poly* staining in liver bile duct structures (Fig. 5e) that also did not colocalise well with spike-AF647 signal (low to absent), also matching data in Figs. 3e and 4c that show enrichment of dACE2 in bile duct. Conversely, in hepatocytes, where we observed and quantified low levels of full-length ACE2 (Figs. 3f and 4c), we see low ACE2*poly* signal that localises with low spike-AF647 signal (Fig. 5f). Overall, the data confirm that spike-AF647 shows low binding at tissue substructures where we previously showed enrichment of dACE2.

## Discussion

With the current consensus suggesting ACE2 is prerequisite for infection of host cells by SARS-CoV-2, we confirmed by immunohistochemistry the expression of ACE2 protein in human kidney, heart, lung, liver, and vasculature (Fig. 1). We observed high levels of staining for ACE2 in the tubules of the renal cortex and lower levels in the glomeruli, and tubules of the renal medulla. In the left ventricle, a critical site for SARS-CoV-2 infection and damage, particularly in patients with Long COVID, all cardiomyocytes examined exhibited ACE2 staining. The lung showed very high levels of ACE2 staining, particularly in airway structures—one of the primary sites exploited by SARS-CoV-2, where it causes the severe acute respiratory syndrome^47^. The hepatocytes of the liver were positive for ACE2, with epithelial cells of bile duct structures showing higher staining levels. Interestingly, the liver is also implicated in SARS-CoV-2 infection, with hepatic impairment seen in patients with severe COVID-19^48^. Positive ACE2 staining in the vasculature also matches previous findings that SARS-CoV-2 induces endothelial dysfunction and dysregulation of immune balance^49^. For a comprehensive review of the organ-specific impacts of COVID-19, see Gavriatopolou et al., 2020^50^.

The emergence of a novel short dACE2 isoform in two independent reports may have important implications in COVID-19 research^45,46^. The isoform is enzymatically inactive, and the spike binding domains of the full-length ACE2 protein are absent, which suggests dACE2 does not bind SARS-CoV-2 spike protein. It is still unclear what the precise effects of such an isoform are in both physiology and in relation to SARS-CoV-2 infection and Long COVID, but the ratio of dACE2 versus the full-length protein may be a contributing factor in the wide inter-individual variation in response to COVID-19. Why some patients are asymptomatic and others suffer severe, long-lasting pathology in response to SARS-CoV-2 remains incompletely understood, and dACE2 should be characterised as part of understanding this phenomenon. We used antibodies raised against different epitopes of the ACE2 protein (ACE2*poly* to multiple epitopes of the extracellular domain and ACE2*mono* to a single epitope between positions 200–300) to distinguish between full-length ACE2 and dACE2 in peripheral organs. In the kidney and heart, colocalisation of the two antibodies in all regions expected to stain for ACE2 indicated that the protein in these tissues was most likely the full-length isoform. In the lung however, we report an enrichment of the dACE2 isoform in the airway epithelia, indicated by comparatively lower ACE2*mono* antibody staining versus ACE2*poly*. Previous findings show high mRNA for dACE2 in the lung, together with the corresponding protein in fully differentiated primary nasal and bronchial epithelial cultures^46^, but we believe we are the first to our knowledge to confirm the presence of dACE2 in native human tissue. In the liver, whilst hepatocytes displayed good colocalisation between ACE2*poly* and ACE2*mono* antibodies, we show expression of dACE2 was enriched in the epithelial cells of bile duct structures. It is interesting that enrichment of dACE2 appears to be predominantly restricted to epithelial cells in our work and that of others. We did not explore ACE2 isoforms in the brain or central nervous system as the Human Protein Atlas suggests that ACE2 mRNA is very low or below the level of detection in human brain^51^.

Quantification of fluorescent imaging confirmed that ACE2 protein was significantly higher in the tubules of the renal cortex versus the renal medulla (p < 0.0001), but there was no significant difference between the two antibodies in the cortical tubules suggesting that full-length ACE2 is the predominant isoform. Glomeruli staining showed no significant difference versus medulla, indicating low ACE2 in these structures. In the lung, ACE2*poly* staining in airways was significantly higher than ACE2*poly* staining in connective tissue (p < 0.0001), and was significantly higher than ACE2*mono* in the airways (p < 0.0001) which was not significantly higher in the airways versus the connective tissue. Blood vessels in the lung showed staining with both antibodies that was significantly higher than that seen in the connective tissue (p < 0.0001 for both), but neither antibody was significantly enriched compared to the other in the blood vessels, indicating good colocalisation and suggesting full-length ACE2 predominated in these structures. In the liver, the qualitative enrichment of dACE2 in the bile duct epithelium was confirmed by quantification, where ACE2*poly* staining was significantly higher than ACE2*mono* (p < 0.0001). When compared to hepatocytes, ACE2*mono* staining showed no significant difference in the bile duct. As with the lung, both antibodies in blood vessels in the liver showed positive staining, which was significantly higher (p < 0.0001 and p < 0.01 for ACE2*poly* and ACE2*mono*) than in the hepatocytes and, again, showed colocalisation.

Lastly, we confirmed low binding of a fluorescently tagged spike-AF647 protein monomer in the airway and bile duct epithelia of the lung and liver respectively, where we saw enriched dACE2. This lies in accordance with the original studies that identified dACE2 as lacking spike binding sites and unable to bind the spike receptor binding domain^45,46^. It is interesting that low spike-AF647 binding is observed in tissue structures recognised as primary targets for SARS-CoV-2 infection and damage, but does point to the ratio of full-length ACE2 and dACE2 in such regions having importance in the range and extent of responses to SARS-CoV-2 reported in COVID-19 patients. It is currently unknown whether enriched dACE2 is beneficial or detrimental in COVID-19. Given that females are less susceptible to SARS-CoV-2 infection and symptoms, despite having higher gene dosing of ACE2^42^, it may be the case that low ACE2, or indeed a ratio shifted towards dACE2, is harmful in COVID-19 outcome, particularly relating to cardiovascular pathology. Conversely, the enrichment of an ACE2 isoform that does not bind SARS-CoV-2 spike protein may be protective. Future studies will clearly be required to delineate the impact of dACE2 in both health and disease.

## Conclusions

COVID-19 patients have displayed great inter-individual variation in susceptibility, and severity of symptoms, and there are likely to be a number of reasons for this. These include initial viral load, biological differences (such as age, sex, and genetic variation), lifestyle choices (diet, smoking etc.), underlying co-morbidities illness, and immune capabilities^44,51–53^. Here we confirm the enriched presence of the novel short dACE2 protein isoform, which lacks catalytic activity and SARS-CoV-2 spike binding sites, in epithelia of both the lung and liver—organs that act as entry points and targets for damage by SARS-CoV-2. The comparative expression of full-length ACE2 to short dACE2 is also hypothesised to contribute to inter-individual variation in response to COVID-19, and it will be highly important to consider the role of this protein isoform in disease pathology and treatment.

## Methods

### Human tissue

Surgical samples of human tissue were obtained with informed consent from Royal Papworth Hospital Research Tissue Bank and ethical approval (05/Q104/142) as anonymised samples, with clinical diagnoses provided where applicable for pathological tissue, but further clinical details undisclosed. Heart tissue used in this study was donated by individuals clinically diagnosed with dilated cardiomyopathy. For control tissues, samples were taken from organs suitable for transplantation that were not used for various reasons such as an unavailability of a suitable recipient. Tissues were snap frozen in liquid nitrogen before storage at − 80 °C. Tissue samples from humans (n ≥ 3 individuals) were cut, using a cryostat (− 20 °C to − 30 °C), into 10 μm sections and thaw mounted onto slides before return to storage at − 80 °C. On the day of experimentation, frozen tissue sections were thawed for 20 min at room temperature (21 °C), encircled with a hydrophobic pen, and subsequently rehydrated with PBS.

### Immunohistochemistry

Hydrated tissue sections were washed 3 × with PBS before fixation with buffered (pH 6.9) 4% formaldehyde solution (1.00496; Merck) for 20 min. For spike-AF647 studies, sections were treated with SARS-CoV-2 spike receptor binding motif protein monomer fluorescently tagged at the N-terminal end with a dye structurally similar to Alexa Fluor 647 (spike-AF647; 1 µM) for 30 min at room temperature in the dark before fixation. Following a further 3 × washes with PBS, non-specific binding was blocked with PBS containing 10% donkey sera for 2 h at room temperature. Sections were then incubated overnight at 4 °C with ACE2*poly* primary goat polyclonal antibody to Human ACE2 (AF933; R&D; 1:100) and/or ACE2*mono* primary rabbit monoclonal antibody to a site between 200 and 300 amino acids (N-terminus) of Human ACE2 (ab108252; Abcam; 1:100), depending on the experiment. Primary antibodies were prepared in PBS with 1% donkey sera, 0.1% Tween-20, and 3.3 mg/mL bovine serum albumin, and control sections were treated with the buffer in the absence of the primary antibodies. After incubating for 24 h at 4 °C in the dark, sections were washed 3 × with PBS with 0.1% Tween-20 before incubation with secondary polyclonal Donkey Anti-Goat IgG H&L antibody conjugated to Alexa Fluor 488 (ab150129; Abcam; 1:200) or Donkey Anti-Rabbit IgG H&L antibody conjugated to Alexa Fluor 555 (ab150066; Abcam; 1:200) prepared at 0.01 mg/mL in PBS with 1% donkey sera, 0.1% Tween-20, and 3.3 mg/mL bovine serum albumin, for 1 h at room temperature. Tissue sections were washed again 3 × with PBS before incubation with Hoechst 33342 nuclear stain (H3570; Invitrogen) prepared at 10 μg/mL in PBS for 15 min at room temperature in the dark. After a final 3 × washes with PBS, slides were blotted dry with lint-free tissue, mounted with ProLong Gold Antifade Mountant (P36930; Invitrogen), covered with a cover slip, and left overnight at room temperature in the dark to dry.

### Slide Scanner Axio Scan.Z1 (Zeiss) imaging

Automated fluorescent images (16 bit, 0.325 × 0.325 μm scaling per pixel) were acquired using a Slide Scanner Axio Scan.Z1 (Zeiss) microscope with a Plan-Apochromat 20×/NA0.8 M27 objective lens connected to a Hamamatsu Orca Flash camera. The system uses an LED light source, which provides more consistent illumination over time. After using bright field imaging to find tissue on the slides, three fluorescent channels were used, with low exposure times to minimise bleaching of the sample. The first (blue channel) used an LED-Module 385 nm light source set at 10% intensity and 10 ms exposure time to illuminate the Hoechst nuclear stain (max excitation and emission of 361 and 497 nm respectively). The second (green channel) used an LED-Module 475 nm light source set at 40% intensity and 20 ms exposure time to illuminate the Donkey Anti-Goat IgG H&L antibody conjugated to Alexa Fluor 488 (max excitation and emission of 490 and 525 nm respectively). The third channel (orange channel) used an LED-Module 567 nm light source set at 80% intensity and 30 ms exposure time to illuminate the Donkey Anti-Rabbit IgG H&L antibody conjugated to Alexa Fluor 555 (max excitation and emission of 555 and 580 nm respectively). Acquired images were saved and visualised using Zeiss ZEN 2 (blue edition) version 3.1.0.0000 (https://www.zeiss.com/microscopy/int/products/microscope-software/zen-lite.html) and/or Orbit Image Analysis^54^ version 3.64 (https://www.orbit.bio/) software.

### Slide Scanner Axio Scan.Z1 (Zeiss) quantification

Acquired fluorescent tissue section images were quantified using Zeiss ZEN 2 (blue edition) software. Regions of interest, such as tubules in the renal cortex, glomeruli, airway epithelia, and bile duct epithelia were identified and a free-hand drawing tool was used to encircle sample regions in fluorescent images (Supplementary Fig. 1). Mean fluorescence intensities in regions of interest were provided by Zeiss ZEN 2 (blue edition) software (Supplementary Fig. 2) for the Donkey Anti-Goat IgG H&L antibody conjugated to Alexa Fluor 488 and Donkey Anti-Rabbit IgG H&L antibody conjugated to Alexa Fluor 555 as markers of full-length ACE2 and short *delta*ACE2 respectively. Due to inherent differences in the brightness of each antibody, mean fluorescence intensities were normalised for each antibody relative to their respective brightness in regions of studied tissues where signal was deemed to be low (e.g. renal medulla, respiratory connective tissue, liver hepatocytes), and presented as mean fold change from these regions ± s.e.m.

### Data analysis and statistics

Quantitative data are expressed as fold change in mean fluorescence intensity ± s.e.m. Replicates (n numbers) are provided in figure legends. Data were analysed using a one-way ANOVA and Tukey’s correction for multiple comparisons looking for differences in fold change in mean fluorescence intensity in regions of interest versus normalised low-level staining regions, and between the ACE2*poly* and ACE2*mono* antibody signal in each region of interest. A *p* value of < 0.05 was determined as significant. Graphical presentation and statistical analyses were performed using GraphPad Prism version 6.07 for Windows (GraphPad Software, La Jolla, California, USA, www.graphpad.com).

### Multiphoton Microscope Leica TCS SP8 MP imaging

Tissue sections treated with spike-AF647 were imaged using a Leica TCS SP8 multiphoton confocal fluorescent microscope (Leica Microsystems), run with Leica Application Suite X (LAS X) acquisition and analysis software. Images were acquired using an HC PL APO CS2 63x/NA1.40 oil immersion objective lens. Excitation/emission wavelengths of 405/410–480 nm at a gain of 702.5 V were used for the Hoechst 33342 nuclear stain (channel pseudo-coloured blue); 488/500–550 nm at gain of 702.6 V for ACE2*poly* immune-reactivity (channel pseudo-coloured green); and 633/650–700 nm at a gain of 672.4 V for spike-AF647 (channel pseudo-coloured red). Images (512 × 512 pixels) were processed using FiJi ((FiJi is Just) ImageJ)^55^ version v1.51n (https://imagej.nih.gov/ij/). Scale bars show 50 µm.

### Opera Phenix High Content Screening System (PerkinElmer) methods

To validate the ACE2*poly* and ACE2*mono* antibodies (Supplementary Fig. 3) used in this study, CHO-K1 cells were plated at 10 k/well in CellCarrier-96 Ultra Plates. Cells were transiently transfected with a commercially available Human angiotensin I converting enzyme (peptidyl-dipeptidase A) 2 (ACE2) tagged with GFP cDNA clone (RG208442; OriGene; 10 ng/µL final) using a TransIT-CHO Transfection Kit (MIR 2174; Mirus Bio) as per the manufacturer’s instructions. After 24 h, cells were washed 3 × with HBSS before fixation with buffered (pH 6.9) 4% formaldehyde solution (1.00496; Merck) for 20 min. Following a further 3 × washes with HBSS, non-specific binding was blocked with HBSS containing 10% donkey sera for 2 h at room temperature. Sections were then incubated overnight at 4 °C with ACE2*poly* primary goat polyclonal antibody to Human ACE2 (AF933; R&D; 1:100) or ACE2*mono* primary rabbit monoclonal antibody to a site between 200 and 300 amino acids (N-terminus) of Human ACE2 (ab108252; Abcam; 1:100). Primary antibodies were prepared in HBSS with 1% donkey sera, 0.1% Tween-20, and 3.3 mg/mL bovine serum albumin, and control wells were treated with the buffer in the absence of the primary antibodies. After incubating for 24 h at 4 °C in the dark, cells were washed 3 × with HBSS with 0.1% Tween-20 before incubation with secondary polyclonal Donkey Anti-Goat IgG H&L antibody conjugated to Alexa Fluor 555 (ab150134; Abcam; 1:200) or Donkey Anti-Rabbit IgG H&L antibody conjugated to Alexa Fluor 555 (ab150066; Abcam; 1:200) prepared at 0.01 mg/mL in HBSS with 1% donkey sera, 0.1% Tween-20, and 3.3 mg/mL bovine serum albumin, for 1 h at room temperature. Cells were washed again 3 × with HBSS before incubation with Hoechst 33,nuclear stain (H3570; Invitrogen) prepared at 10 μg/mL in HBSS for 15 min at room temperature in the dark. After a final 3 × washes with HBSS, cells were maintained in 100 µL HBSS. Fluorescent confocal images of cells were acquired using the Opera Phenix High Content Screening System (PerkinElmer) microscope with a 40×/NA1.1 water immersion objective. Excitation/emission laser and filter sets for three fluorescent channels were used: 405/435–480 nm (blue) for the Hoechst 33342 nuclear stain, 488/500–550 nm (green) for GFP, and 561/570–630 nm (yellow) for the Donkey Anti-Goat IgG H&L antibody conjugated to Alexa Fluor 555 (ab150130; Abcam) or Donkey Anti-Rabbit IgG H&L antibody conjugated to Alexa Fluor 555 (ab150066; Abcam). Laser intensities were set to 50% transmission with a 50 ms exposure time for all channels. Scale bars show 50 μm unless specified otherwise.

## Supplementary Information

Supplementary Information.

## Data Availability

Data available on request.
