# Supplementary material for: Differential expression in humans of the viral entry receptor ACE2 compared with the short deltaACE2 isoform lacking SARS-CoV-2 binding sites
Source: Sci Rep. 2021 Dec 21;11:24336. doi: 10.1038/s41598-021-03731-9 (PMC8692523; doi:10.1038/s41598-021-03731-9)
Supplement: Supplementary file 1 — Supplementary Information. [file 41598_2021_3731_MOESM1_ESM.docx]

**Differential expression in humans of the viral entry receptor ACE2 compared with the short *delta*ACE2 isoform lacking SARS-CoV-2 binding sites**

Thomas L. Williams^1^, Gregory Strachan^2^, Robyn G.C. Macrae^1,3^, Rhoda E. Kuc^1^, Duuamene Nyimanu^1^, Anna L. Paterson^4^, Sanjay Sinha^3^, Janet J. Maguire^1§^, Anthony P. Davenport^1§^*

1. Experimental Medicine and Immunotherapeutics, University of Cambridge, Addenbrooke’s Hospital, Cambridge, U.K.

2. Wellcome Trust-MRC Institute of Metabolic Science, Metabolic Research Laboratories, Addenbrooke’s Biomedical Campus, Cambridge, U.K.

3. Wellcome-MRC Cambridge Stem Cell Institute, Jeffrey Cheah Biomedical Centre, University of Cambridge, Cambridge, U.K.

4. Department of Pathology, Royal Papworth Hospital NHS Foundation Trust, Cambridge University Hospitals NHS Foundation Trust

^§^Joint senior authors

Author for Correspondence:

*Anthony P. Davenport

Experimental Medicine and Immunotherapeutics,

University of Cambridge,

Level 6, Addenbrooke’s Centre for Clinical Investigation,

Box 110, Addenbrooke's Hospital, Cambridge, CB2 0QQ, U.K.

Tel: +44(0)1223 336899

[apd10@medschl.cam.ac.uk](mailto:apd10@medschl.cam.ac.uk)

Correspondence should be addressed to A.P.D. ([apd10@medschl.cam.ac.uk](mailto:apd10@medschl.cam.ac.uk))

**Supplemental Information**

**
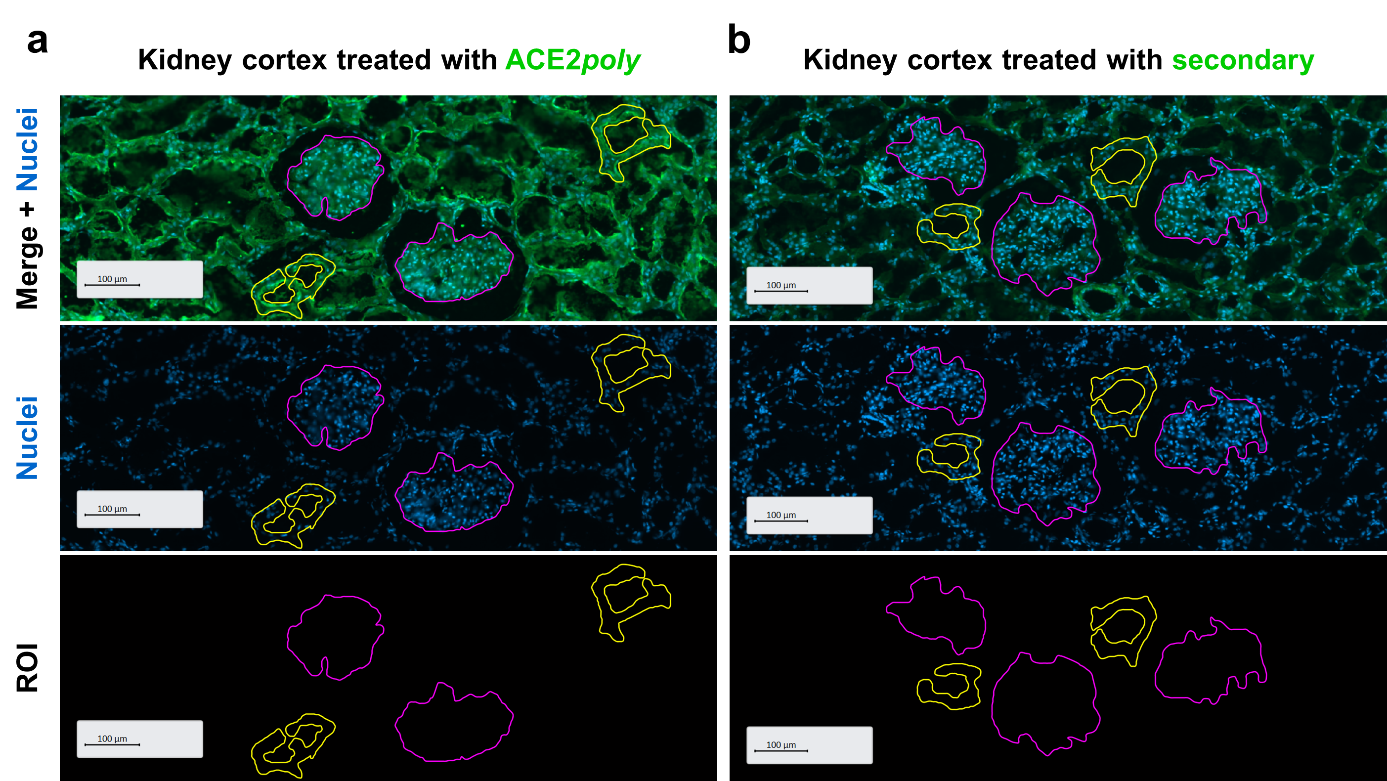
**

**Supplementary Figure 1.** Representative examples of regions of interest (ROI) for quantification of ACE2 fluorescence in human tissue sections using ZEN software (Zeiss). Scale bars show 100 µm. **a** (Left column) positive immunohistochemical staining of ACE2 protein in human kidney cortex with the ACE2*poly* visualised using a secondary antibody conjugated to Alexa Fluor 488 (shown in green). A free-hand drawing tool was used to isolate glomeruli (magenta) or tubules of the renal cortex (yellow) as ROI. Note distinct clusters of nuclear staining in glomeruli (middle row). Mean fluorescence intensity (measured in grayscales at a bit depth of 16) could then be provided for individual ROI. Note that the lumen of tubular structures are discounted from the analyses to prevent inaccurate averaging of fluorescence intensity. **b** (Right column) control human kidney cortex treated with the secondary antibody conjugated to Alexa Fluor 488 in the absence of ACE2*poly*. Control tissue showed less staining for ACE2. Again, glomeruli are isolated in magenta, and tubules in yellow, and mean fluorescence intensities in these ROI could be provided.


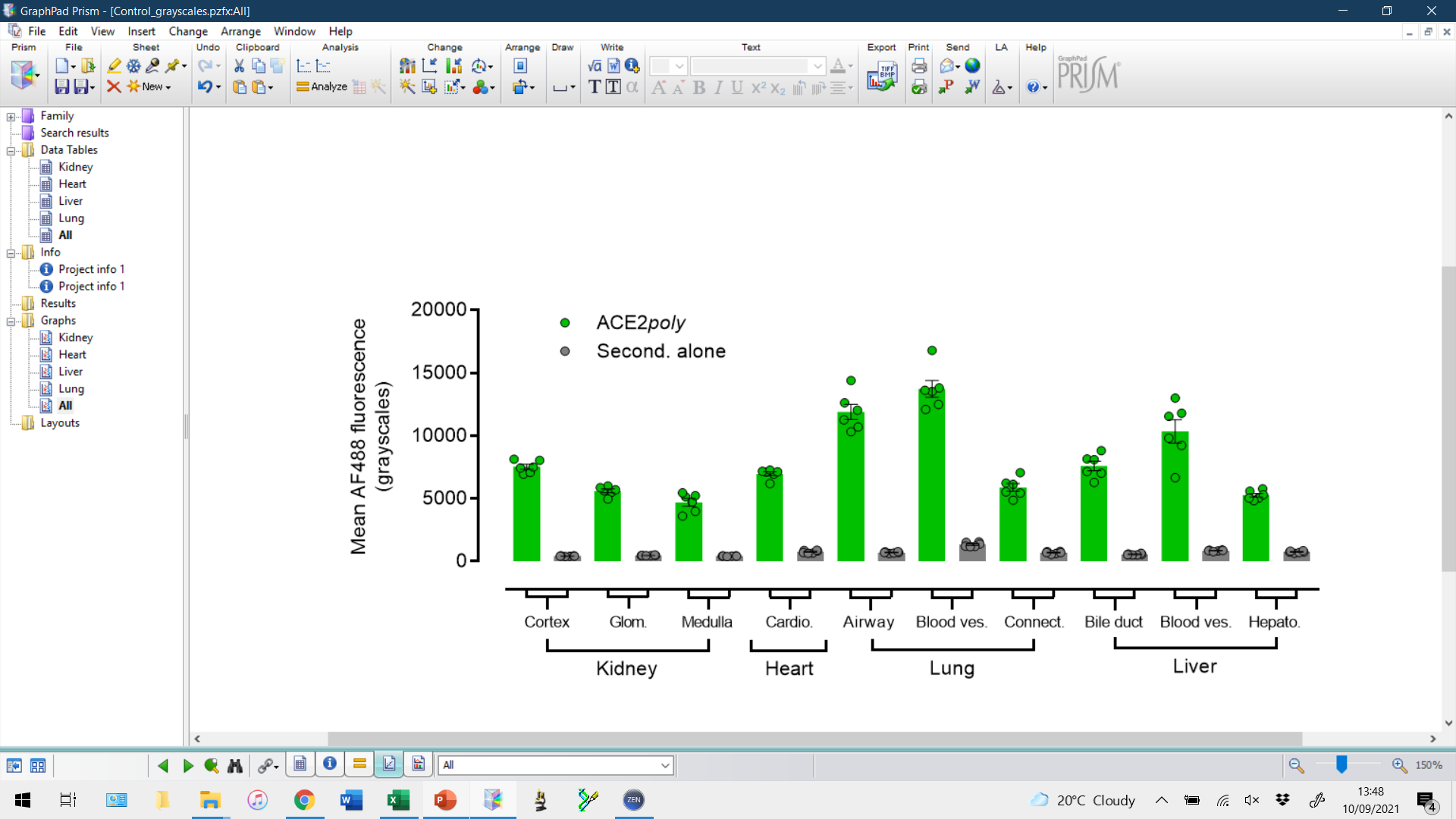


**Supplementary Figure 2.** Bar chart (mean ± s.e.m. with individual data points) showing quantification of mean fluorescence intensity (measured in grayscales at a bit depth of 16) for ACE2 fluorescence in regions of interest (ROI) in a panel of human tissue sections (n = 6 ROI; across n ≥ 2 tissue donors). ROI were identified as described in Supplementary Fig. 1. For each ROI, positive staining with ACE2*poly* (shown in green) in all tissue types was significantly higher than in the respective ROI in tissue treated with the secondary antibody in the absence of ACE2*poly* (Second. alone; shown in grey). Significance (p < 0.05) was determined using a one-way ANOVA with Tukey’s correction for multiple comparisons. For clarification, Glom. = glomeruli; Cardio. = cardiomyocytes; Blood ves. = blood vessels; Hepato. = hepatocytes.


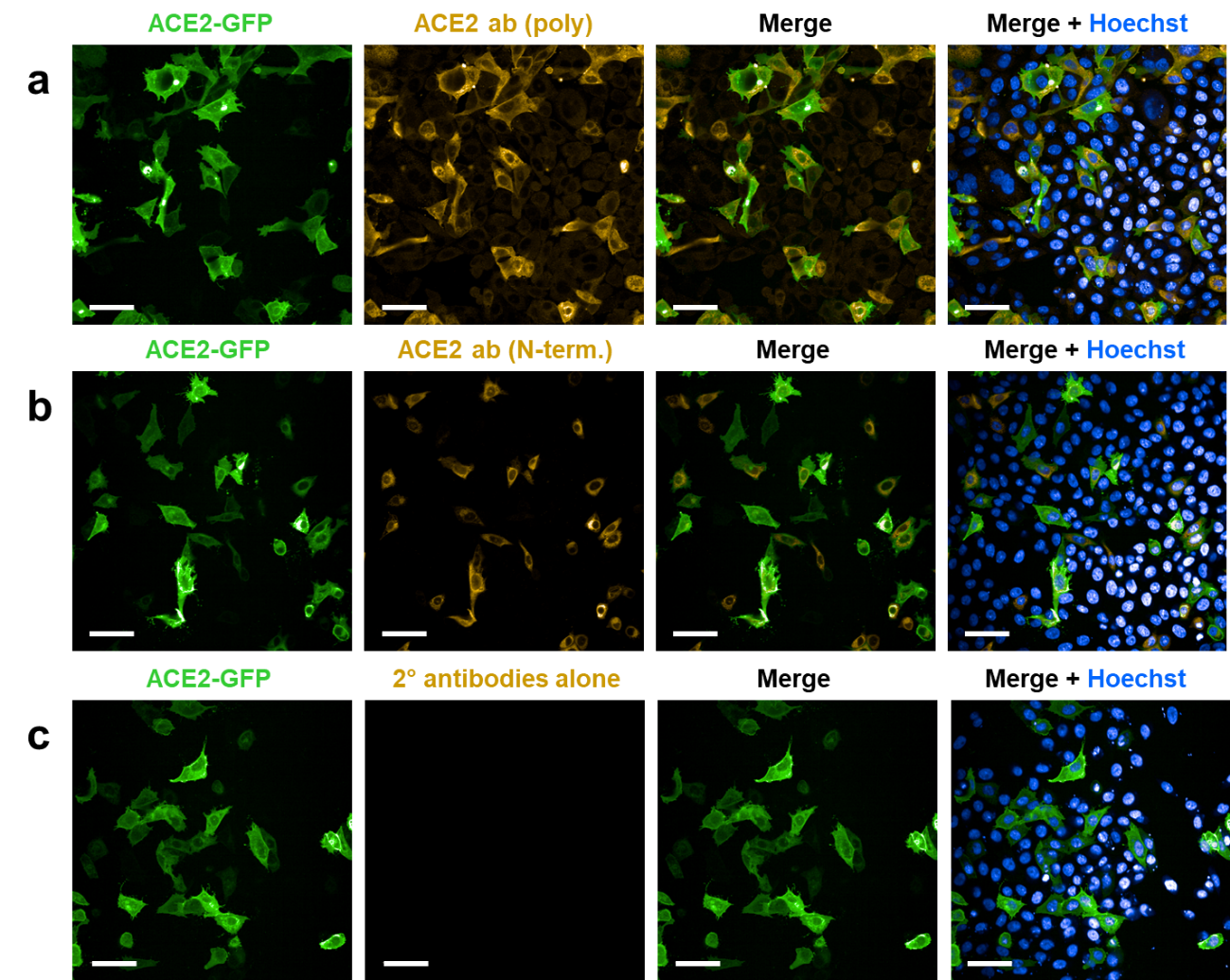


**Supplementary Figure 3.** Validation of ACE2 specific antibodies in CHO-K1 cells. **a-c** Representative fluorescent confocal images (n=3 experiments performed in duplicate) of CHO-K1 cells transiently expressing an ACE2 construct tagged at the C-terminus with GFP, fixed in 4% formaldehyde and treated with ACE2*poly* or ACE2*mono*, or the secondary antibodies alone, as indicated in the figure. Merged images show fluorescent channels for GFP (488 nm) and the secondary antibodies (555 nm) used to visualise primary antibody staining. Merged images in the far right column include Hoechst 33342 nuclear stain (405 nm). Scale bars show 50 μm. **a** ACE2*poly* binds specifically to only those CHO-K1 cells expressing the ACE2-GFP construct, as indicated in the merge. Hoechst reveals the portion of cells that have not taken up and/or do not express the ACE2-GFP plasmid, that also do not stain with ACE2*poly*. **b** ACE2*mono* also binds specifically to only those CHO-K1 cells expressing the ACE2-GFP construct, as indicated in the merge. Again, Hoechst reveals the portion of cells that have not taken up and/or do not express the ACE2-GFP plasmid, that also do not stain with ACE2*mono*. **c** Secondary antibodies alone do not stain any cells in the observed population, including those expressing the ACE2-GFP construct, indicating staining requires specific binding of the primary antibodies to ACE2.
